# Big data analytics for CLEC5A dynamics based on single cell genomics and proteomics reveal its diverse functions in human diseases

**DOI:** 10.7150/ntno.135045

**Published:** 2026-07-30

**Authors:** Xiaoyuan Bai, Jingke Yao, Sarah Abdallah, Hongye Huang, Shijie Liu, Zhicheng Jin, Bingzhong Xue, Hang Shi, Zufeng Ding

**Affiliations:** 1 Department of Biology, Georgia State University, Atlanta, GA, 30303, USA.; 2 Department of Chemistry, Georgia State University, Atlanta, GA, 30303, USA.

**Keywords:** CLEC5A, big data analytics, scRNA-seq, congenital heart disease

## Abstract

**Background:**

CLEC5A (C-type lectin domain family 5 member A) is an innate immune receptor implicated in inflammatory signaling, contributing to hyperinflammatory responses in infections and sterile inflammation. However, CLEC5A dynamics in human diseases remain to be identified. Here, we systematically characterized CLEC5A dynamics in humans across cells, tissues, and disease states, and to explore the functional significance of CLEC5A in macrophage activation based on single-cell genomics.

**Methods:**

With multi-omics (scRNA-seq, proteomics and big data analytics), we analyzed extensive human transcriptomic datasets (>42,000 samples) to profile CLEC5A expression by cell type, tissue, and disease. Single-nucleus RNA-seq (snRNA-seq) from pediatric congenital heart disease and a virtual CLEC5A gene knockout were also performed to characterize CLEC5A dynamics in humans.

**Results:**

CLEC5A is highly enriched in innate immune cells, particularly in macrophages and neutrophils. Baseline CLEC5A in most tissues is low, but it is markedly upregulated in inflammatory and infectious diseases. CLEC5A expression has sex-specific differences in certain organs. Single-cell analysis showed that CLEC5A can be considered novel marker of proinflammatory macrophages with elevated cytokine production, antigen presentation, and impaired phagocytosis. Virtual CLEC5A knockout analysis identified coordinated perturbation of immune-regulatory pathways and overlapping genes linking CLEC5A to macrophage activation networks.

**Conclusion:**

CLEC5A is predominantly expressed in myeloid cells and acts as a key amplifier of inflammation in human diseases. Our findings highlight CLEC5A as a potential biomarker and therapeutic target in myeloid-driven hyperinflammatory conditions, warranting further experimental and translational validation.

## Introduction

C-type lectin domain family 5 member A (CLEC5A), also known as myeloid DAP12-associating lectin-1 (MDL-1), is a DAP12-associated pattern recognition receptor^1^. Clec5a is predominantly expressed on myeloid-lineage cells, including macrophages, monocytes, neutrophils, and dendritic cells^2^,^3^. Through association with the adaptor protein TYROBP (DAP12), CLEC5A transduces activation signals via spleen tyrosine kinase (Syk) and downstream signaling of phosphatidylinositol 3-kinase (PI3K), Akt, and nuclear factor κB (NF-κB) pathways, thereby amplifying innate immune activation^4^. CLEC5A has been implicated in the pathogenesis of several viral infections^5^, including dengue, Japanese encephalitis, influenza, and COVID-19^6^-^9^. In these contexts, CLEC5A activation in macrophages and neutrophils contributes to cytokine storm, vascular leakage, and organ damage, whereas CLEC5A inhibition attenuates inflammation response and improves cell survival^10^. Beyond infection, CLEC5A has been associated with autoimmune and chronic inflammatory conditions such as arthritis^11^, chronic obstructive pulmonary disease (COPD)^12^,^13^, and adult-onset Still's disease^14^, where its expression correlates positively with disease activity and circulating proinflammatory cytokines. These findings identify CLEC5A as a key amplifier of myeloid-driven inflammation and a potential therapeutic target in hyperinflammatory disorders. In this study, CLEC5A-associated hyperinflammation refers to an excessive and dysregulated innate immune response characterized by myeloid-cell activation, elevated pro-inflammatory cytokines, inflammasome activation, vascular leakage, and tissue injury^15^. This process may involve multiple organ systems, including cardiovascular, pulmonary, neurological, hepatic, renal, and immune systems. Increasing evidence indicates that in human and murine atherosclerosis, CLEC5A is highly expressed in lesional macrophages^16^,^17^, and the expression is induced by pathophysiologic stimuli such as oxidized low-density lipoprotein (ox-LDL) and hypoxia^18^,^19^. These findings raise the possibility that CLEC5A may represent a novel signaling in cardiometabolic diseases.

Despite the above advances, a comprehensive understanding of CLEC5A expression across human tissues and disease states remains to be identified. The previous studies in CLEC5A mainly focused on specific immune cell subsets or individual organ systems^20^. Therefore, we conducted comprehensive big data analytics of Clec5a based on proteomics, bulk and single-nucleus RNA-seq (snRNA-seq) datasets in humans. By integrating transcriptomic profiles from a wide range of human tissues and disease states, we characterize the cellular, developmental, and tissue-specific distribution of Clec5a, with emphasis on immune and metabolically active systems. Our findings for the first time reveal extensive and context-dependent dysregulation of Clec5a across immunity and metabolism as well as a variety of tissues and disease states, also highlighting developmental and sex-related differences. In addition, our study for the first time incorporated a macrophage-focused snRNA-seq analysis^21^ and complementary functional predictions for the deepened insight into CLEC5A's dynamics. By examining CLEC5A expression at single-cell resolution in immune cells and performing in virtual knockout experiments^22^, we aimed to link CLEC5A to specific cellular programs and reveal its complicated network-level impacts on human diseases. These approaches provide a granular understanding of CLEC5A's function, supplementing the findings with cell-type specificity and mechanistic predictions. Overall, our big data analytics will be the first study exploring the diverse role of Clec5a in human diseases, providing compelling evidence that Clec5a is a promising therapeutic target in cardiovascular and other human inflammation-dependent diseases.

## Results

**Figure 1:**
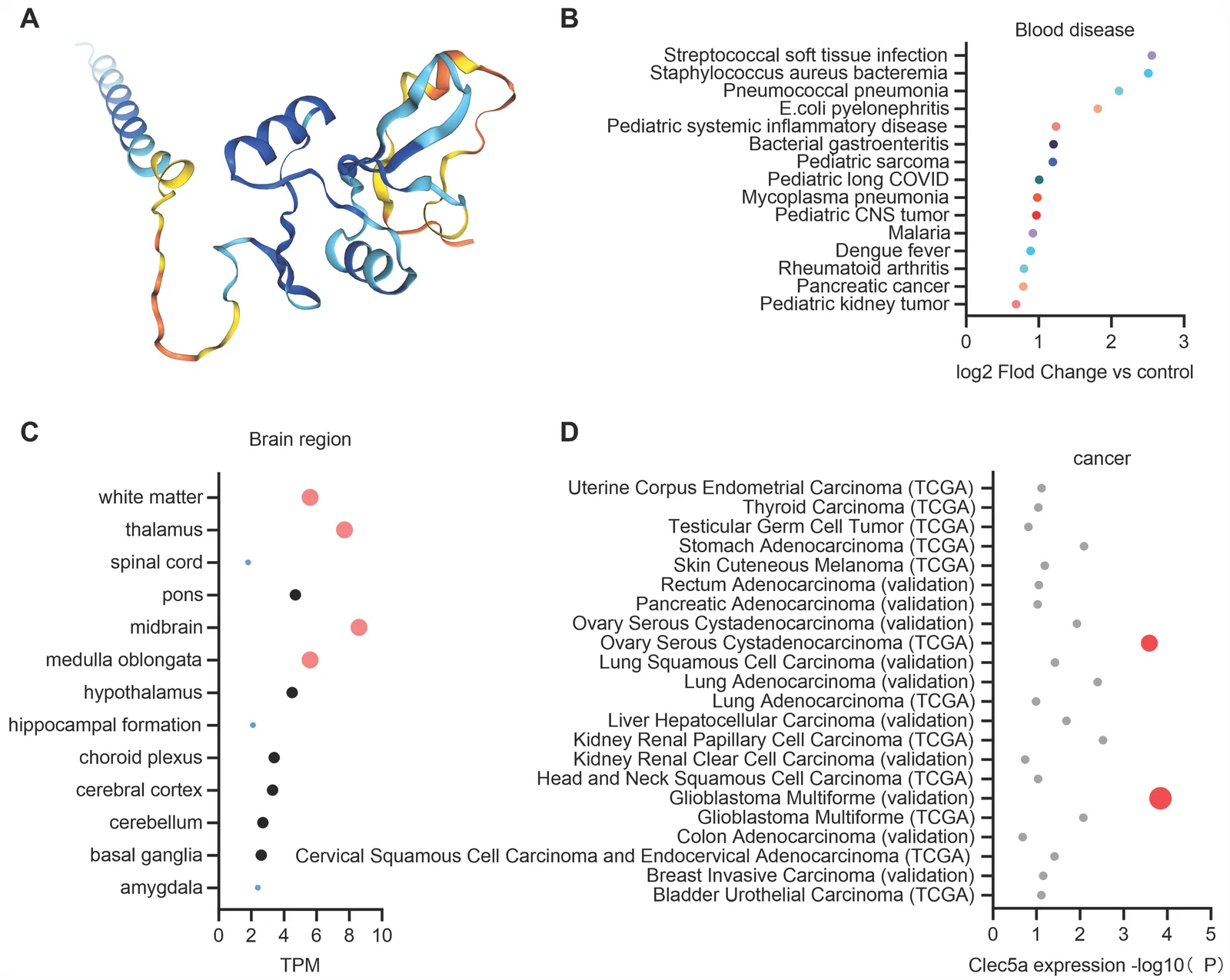
** Big data analytics for CLEC5A expression at protein levels in humans.** (**A**) Predicted three-dimensional structure of human CLEC5A protein, with dark blue representing the highest confidence and orange the lowest confidence. (**B**) CLEC5A expression changes in blood diseases. (**C**) CLEC5A transcript expression levels in brain. (**D**) CLEC5A expression in multiple human cancer types. The data was from the database of the Human Protein Atlas. The data were analyzed with GraphPad Prism 10.1.2.

**Big data analytics for Clec5a expression patterns in humans.** To characterize the global expression landscape of CLEC5A in humans, we performed big data analytics using publicly available transcriptomic and proteomic datasets based on the Human Protein Atlas^23^. **Figure 1A** shows the predicted structure of CLEC5A colored according to a per-residue confidence score^24^. Proteomic profiling of blood diseases showed that CLEC5A protein levels were markedly increased in a widely profile of infectious and inflammatory conditions, including bacterial infections and systemic inflammatory disorders, highlighting its close association with immune activation and inflammatory responses (**Figure 1B**).Transcriptomic analysis revealed that CLEC5A is expressed at low to moderate levels across human brain regions, with relatively higher expression in the midbrain, thalamus, and medulla oblongata, and minimal expression in neuron-rich regions such as the hippocampus and amygdala (**Figure 1C**).Prognostic analysis based on TCGA and validation datasets revealed that CLEC5A expression is significantly associated with patient outcomes in a subset of human cancers^25^. In several tumor types, the CLEC5A expression elevated has no significant difference, whereas it was associated with favorable outcomes in some cancers (**Figure 1D**). These findings suggest a potential role for CLEC5A in tumor-associated immune or inflammatory processes rather than tumor cell-intrinsic expression.

Together, integrative transcriptomic and proteomic analyses indicate that CLEC5A is predominantly linked to immune- and inflammation-related biological contexts across human tissues and diseases, providing a strong rationale for subsequent cell type-resolved analyses to define its cellular sources and functional relevance in chronic inflammatory heart disease.

**Figure 2:**
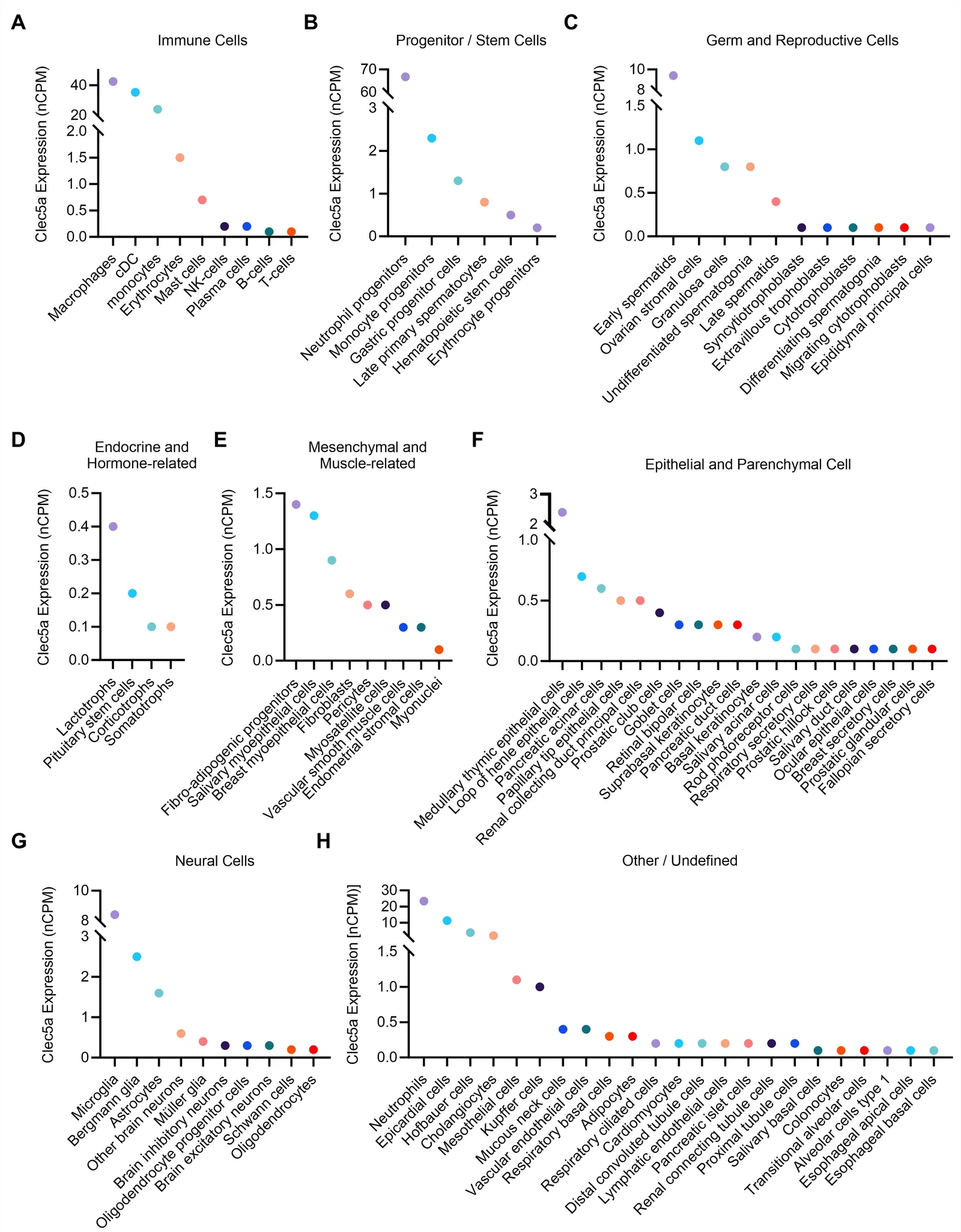
** Single-cell transcriptomics for CLEC5A expression across a variety of human cell types** (**A-H**). The data of single-cell transcriptomics was downloaded from the Human Protein Atlas Cell Type resource, which compiles normalized expression data (nCPM) aggregated across 154 annotated cell types from 36 single-cell RNA-seq datasets. The data were analyzed by GraphPad Prism (10.1.2).

**RNA-seq big data analytics for Clec5a expression in human cells.** Clec5a is known to enhance inflammatory cytokine production during infection and sterile injury^26^. To identify the cellular sources of Clec5a in humans, we performed second big data analytics for its transcript levels across a broad range of human cell types^27^. Innate immune cells are central initiators of inflammatory responses^28^. Clec5a expression is strongest in innate immune cells while macrophages have the highest transcript abundance. Conventional dendritic cells and monocytes also displayed robust expressions of Clec5a. Other myeloid related cells, including erythrocytes and mast cells, express Clec5a at lower but detectable levels. Plasma cells, B cells, T cells and natural killer cells showed minimal expression of Clec5a. These findings indicate a predominant role of Clec5a in innate immune activation rather than in adaptive immunity (**Figure 2A**). Progenitor and stem cells maintain hematopoietic output and generate mature immune cells^29^,^30^. Neutrophil progenitors expressed the highest levels within this category and a variety of progenitors expressed Clec5a, including monocyte progenitors, gastric progenitor cells, late primary spermatocytes, hematopoietic stem cells, erythrocyte progenitors (**Figure 2B**). Germ and reproductive cells show variable expressions of Clec5a. Early spermatids show the highest levels of Clec5a expression. Other germ and reproductive cells express at moderate or minimal levels, including ovarian stromal cells, granulosa cells, late spermatids, cytotrophoblasts, epididymal principal cells. These observations indicate that Clec5a activity in reproductive tissues is higher at early germline populations (**Figure 2C**).

Endocrine related mesenchymal cells 31,32 (e.g., lactotroph cells, pituitary stem cells, corticotrophs and somatotrophs) demonstrate minimal expression of Clec5a (**Figure 2D**). Mesenchymal and muscle related stromal cells provide mechanical support and participate in tissue remodeling^33^,^34^. Fibro-adipogenic progenitors, salivary myoepithelial cells, breast myoepithelial cells, fibroblasts, pericytes and myoepithelial cells show detectable levels of Clec5a. Vascular smooth muscle cells, endometrial stromal cells and myonuclei express Clec5a at lower levels (**Figure 2E**). Epithelial and parenchymal cells carry out barriers, metabolic and secretory functions. Across these lineages, Clec5a expression remains at a low level^35^. Other epithelial and parenchymal cells, including salivary duct cells, prostatic hillock cells, ocular epithelial cells, and fallopian secretory cells show low to minimal Clec5a expression (**Figure 2F**). The expression of Clec5a in neural populations demonstrates very limited expression of Clec5a. Microglia has the highest levels of Clec5a within this category but is still lower than classical macrophages. Brain inhibitory neurons, Schwann cells, oligodendrocytes display minimal expression of Clec5a. These findings suggest that Clec5a may have a limited contribution to neural immune regulation (**Figure 2G**). In addition, our analysis showed that Clec5a is also highly expressed in neutrophils, epicardial cells, Hofbauer cells and cholangiocytes (**Figure 2H**).

Overall, Clec5a expression is strongly enriched in innate immune and myeloid-lineage progenitor populations, showing the highest transcript levels in macrophages, monocytes, dendritic cells, neutrophils and neutrophil progenitors. In contrast, Clec5a has a low expression in most germ and reproductive cells, mesenchymal and muscle-related cells, endocrine cells, epithelial and parenchymal cells, and neural populations.

**Figure 3:**
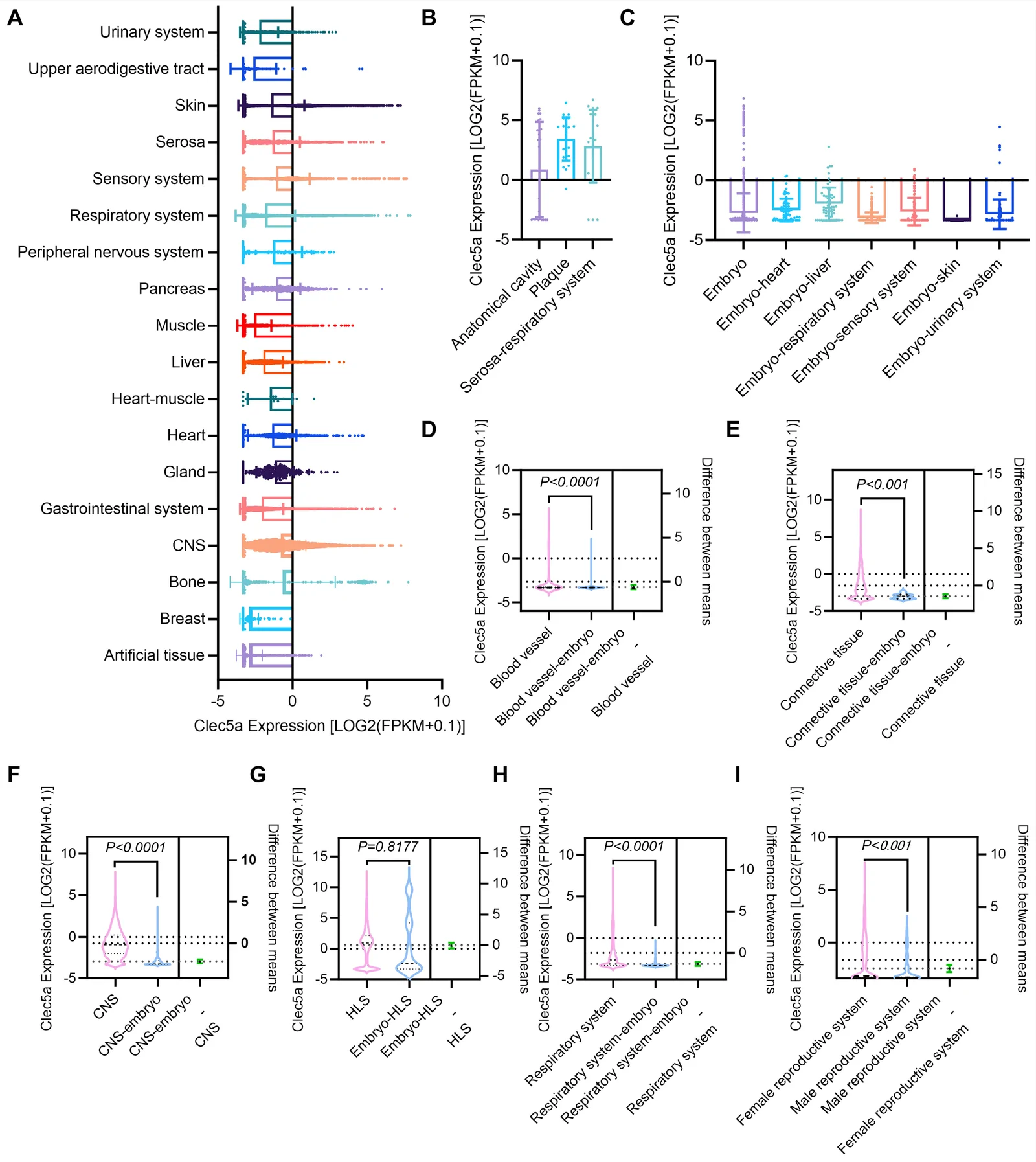
** Clec5a expression in human tissues.** Big data analytics based on RNA-seq or scRNA-seq for Clec5a expression in (**A-C**) human tissues sorted alphabetically, (**D-H**) human tissues from adult and embryo, and (**I**) human reproductive system from female and male. Original data of RNA-seq or scRNA-seq for Clec5a expression, quantified by Log2 (FPKM + 0.1), were downloaded from QIAGEN OmicSoft Land Explorer. The data were analyzed with GraphPad Prism 10.1.2 and shown as the mean ± SD. Dunnett's one-way ANOVA was used for multiple comparisons between disease types and normal control. P < 0.05 was considered statistically significant.

**RNA-seq big data analytics for Clec5a expression in human tissues.** Clec5a is a key inflammatory driver in cardiovascular pathology, including atherosclerosis, myocardial infarction, vascular inflammation, and cardiac remodeling^36^. The activity of Clec5a is also implicated in infection-induced inflammation, metabolic dysregulation, and sterile tissue injury^37^, suggesting that Clec5a contributes broadly to inflammatory processes that influence cardiovascular homeostasis. Therefore, we performed the third big data analytics to assess Clec5a expression across human tissues affected by these diseases^23^. Our data revealed that Clec5a is slightly expressed in numerous human tissues, which are displayed in alphabetically order for clarity. Notably, Clec5a is highly expressed in plaque and serosa-respiratory system (**Figure 3A-B**). Extensive Clec5a expression was also observed in embryonic tissues, including blood vessel, connective tissue, Central nervous system (CNS), hematopoietic and lymphoid system (HLS), and other embryo tissues including heart, liver, respiratory system, sensory system, skin, and urinary system (**Figure 3C**). Notably, Clec5a expression in embryo blood vessel is 34.4 % lower (p < 0.0001) than in normal adult blood vessel (**Figure 3D**). Clec5a expression in embryo connective tissue is 63.4% lower (p < 0.0001) than in normal adult connective tissue (**Figure 3E**). Clec5a expression in embryo CNS is 77.7% lower (p < 0.0001) than in normal adult CNS (**Figure 3F**). In the hematopoietic and lymphoid system, there is no difference (p =0.8177) in Clec5a expression between normal and embryo (**Figure 3G**). Clec5a expression in embryo respiratory system is 59.9 % lower (p < 0.0001) than that in normal adult respiratory system (**Figure 3H**). In the reproductive system, Clec5a expression is 77.6% higher in females (p < 0.0001) than in males (**Figure 3I**).

Together, these findings demonstrate ubiquitous Clec5a transcript expression across human tissues, indicating the Clec5a may influence the function of diverse tissues and contribute to a large variety of human disease processes.

**Figure 4:**
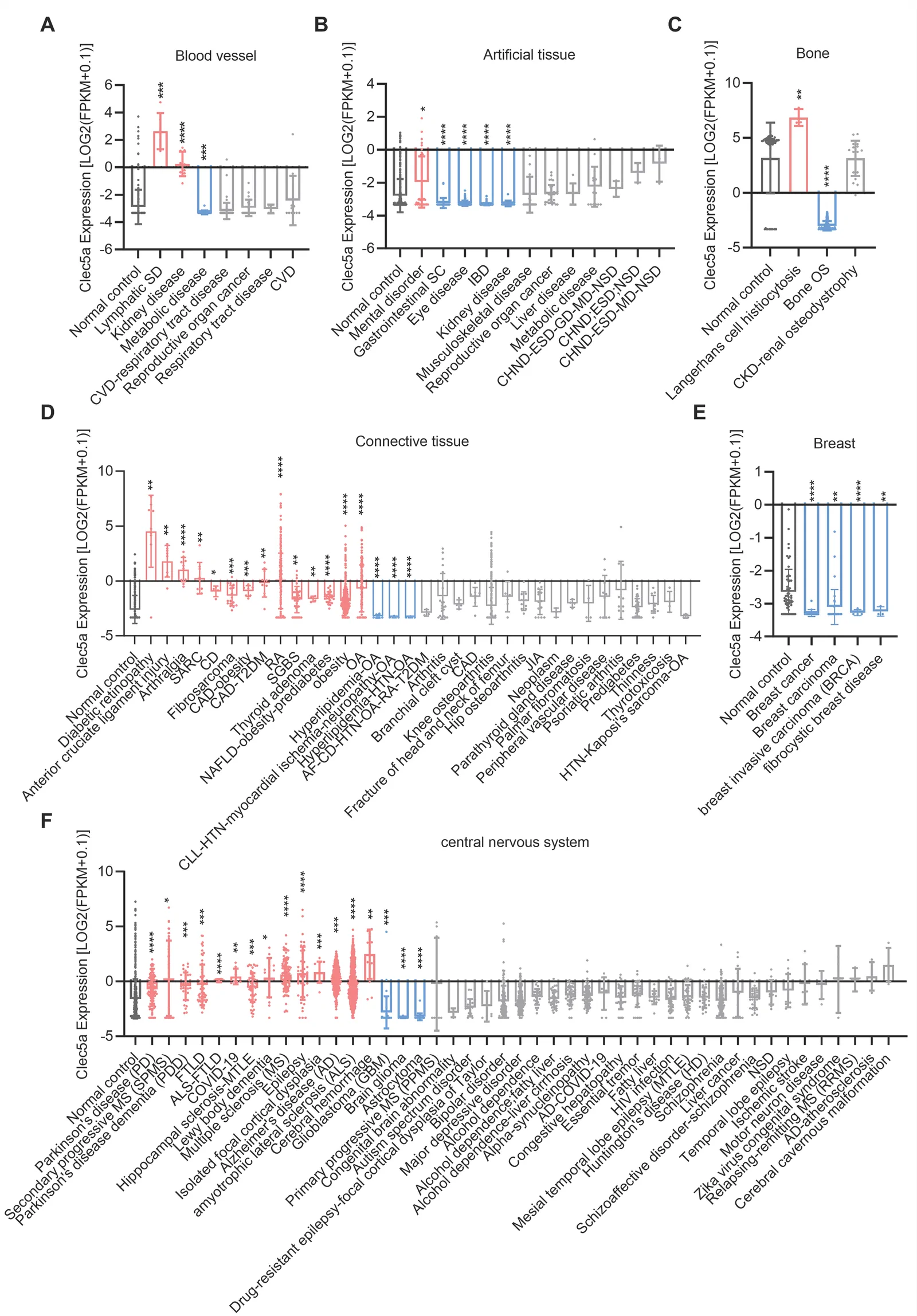
** Clec5a expression in human diseases focusing on specific tissues,** including (**A**) blood vessel, (**B**) artificial tissue, (**C**) bone, (**D**) connective tissue, (**E**) breast, and (**F**) central nervous system. Original data of RNA-seq or scRNA-seq for Clec5a expression, quantified by Log2 (FPKM + 0.1), were downloaded from QIAGEN OmicSoft Land Explorer. The data were analyzed with GraphPad Prism 10.1.2 and shown as the mean ± SD. Dunnett's one-way ANOVA was used for multiple comparisons between disease types and normal control. P < 0.05 was considered statistically significant.

**Clec5a expression in human tissue-specific diseases.** Next, we investigated Clec5a expression in a wide range of human diseases with tissue-specific identification. In blood vessels, compared with normal controls, CLEC5A expression shows a marked and statistically significant increase from patients with kidney diseases (p<0.0001) and lymphatic system diseases(p<0.001) (**Figure 4A**). Artificial tissues are synthetically generated materials designed to replace or integrate with damaged human tissues^38^. In artificial tissue, Clec5a expression is significantly lower in eye disease, gastrointestinal system cancer and IBD (**Figure 4B**). Bone is a mineralized tissue essential for structural support and hematopoiesis. Clec5a expression is higher (p=0.0021) in Langerhans cell histiocytosis but lower in bone Osteosarcoma (OS) (p<0.0001) (**Figure 4C**). Connective tissue supports and links various organs and structures^39^. Clec5a is highly expressed in diseases such as anterior cruciate ligament injury, arthralgia, diabetic retinopathy, obesity, Osteoarthritis (OA), psoriatic arthritis (**Figure 4D**). There is no difference in breast with cancer, carcinoma, breast invasive carcinoma, and fibrocystic breast disease compared with normal control (**Figure 4E**). CNS serves as the central neural hub that receives, integrates, and generates responses to sensory inputs^40^. Common human disorders affecting the CNS include brain tumors, Parkinson's disease, Alzheimer's disease, and stroke. Across CNS disorders, our big data analytics revealed widespread dysregulation of Clec5a expression. Clec5a expression is higher in diseases such as Alzheimer's disease (AD), amyotrophic lateral sclerosis (ALS), cerebral cavernous malformation, cerebral hemorrhage, COVID-19, epilepsy, isolated focal cortical dysplasia, Lewy body dementia, multiple sclerosis (MS), Parkinson's disease, Parkinson's disease dementia (PDD), secondary progressive MS (SPMS). In contrast, expression of Clec5a is lower in brain glioma, glioblastoma (GBM), and astrocytoma (**Figure 4F**).

**Figure 5:**
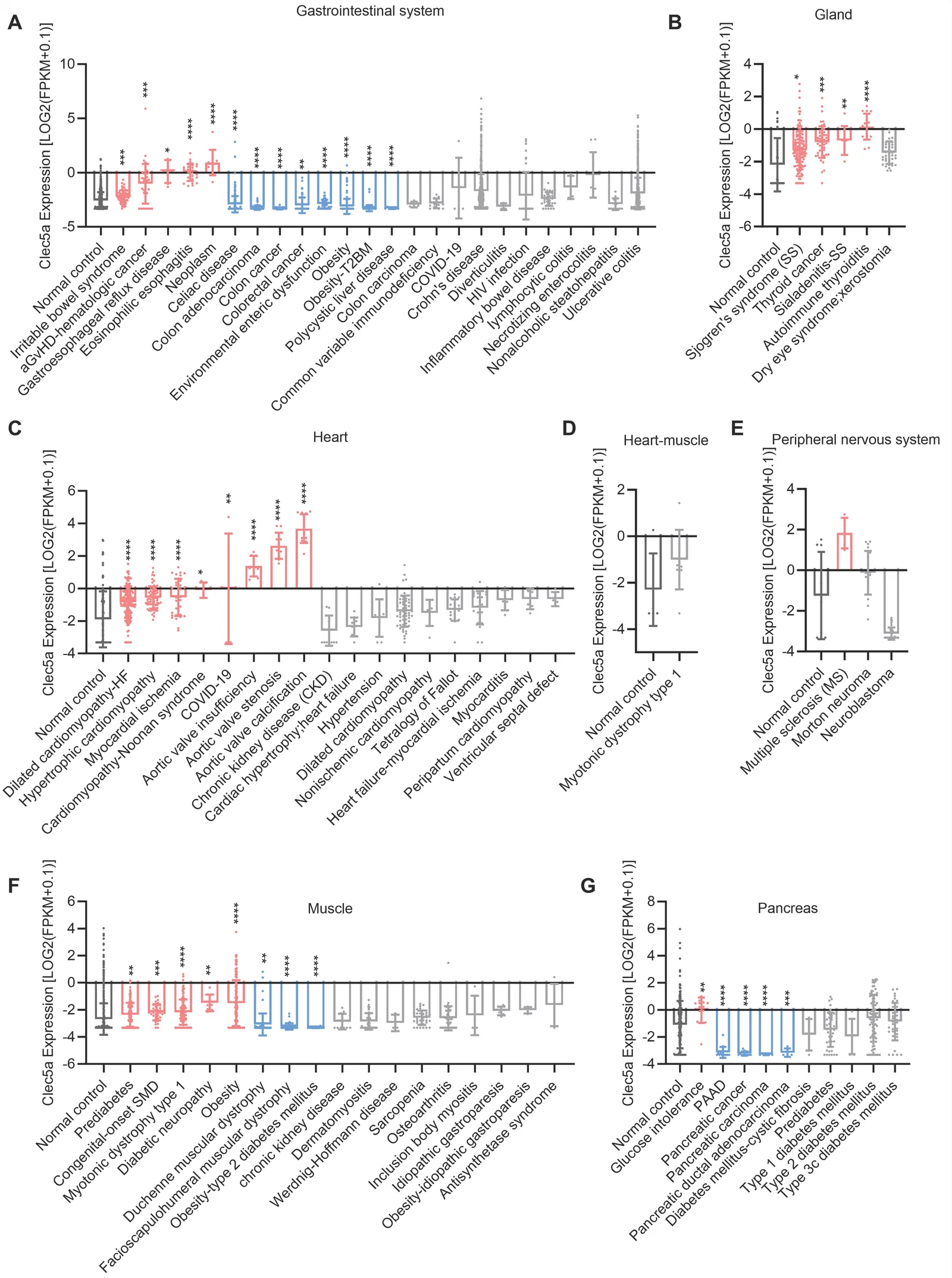
** Clec5a expression in human tissues associated with related diseases**, including (**A**) Gastrointestinal system, (**B**) Gland, (**C**) Heart, (**D**) Heart muscle, (**E**) Peripheral nervous system, (**F**) Muscle, and (**G**) Pancreas. Original data of RNA-seq or scRNA-seq for Clec5a expression, quantified by Log2 (FPKM + 0.1), were downloaded from QIAGEN OmicSoft Land Explorer. The data were analyzed with GraphPad Prism 10.1.2 and shown as the mean ± SD. Dunnett's one-way ANOVA was used for multiple comparisons between disease types and normal control. P < 0.05 was considered statistically significant.

The gastrointestinal system comprises organs responsible for digestion and absorption. We found that Clec5a expression is moderately to highly increased in the gastrointestinal system during related human diseases such as Crohn's disease, gastroesophageal reflux disease, necrotizing enterocolitis, and neoplasm^41^. However, Clec5a is lowly expressed in the gastrointestinal system during celiac disease, colorectal adenocarcinoma, obesity (**Figure 5A**). Glands secrete biologically active substances (e.g., hormones, enzymes, sweat, or saliva) that play important role in maintaining body functions. In gland tissue, we found that, compared with normal control, Clec5a expression is 106.5% higher (p<0.0001) in autoimmune thyroiditis, 34% higher (p=0.0282) in dry eye syndrome xerostomia, 40.8% higher (p=0.0317) in Sjogren's syndrome (SS), 67.8% higher (p=0.0074) in sialadenitis-SS and 63.3% higher (p=0.0005) in thyroid cancer (**Figure 5B**).

In heart, Clec5a expression is significantly higher in aortic valve calcification, aortic valve insufficiency, aortic valve stenosis, cardiomyopathy-Noonan syndrome, COVID-19, dilated cardiomyopathy-heart failure, hypertrophic cardiomyopathy, myocardial ischemia (p<0.0001) (**Figure 5C**). However, there is no difference in Clec5a expression between normal and myotonic dystrophy type 1 group from cardiac muscle (**Figure 5D**).

The peripheral nervous system transmits signals between the CNS and the body^42^. In peripheral nervous system, we found that, compared with normal control, Clec5a expression is markedly higher (p<0.0001) in multiple sclerosis. In contrast, Clec5a expression is lower (p<0.0001) expressed in neuroblastoma (**Figure 5E**). Muscle disorders are conditions causing muscle weakness that play crucial roles in regulating cardiac, skeletal, or smooth muscle functions. Clec5a expression is 44.6% higher (P=0.0080) in diabetic neuropathy, 18.9% higher (p<0.0001) in myotonic dystrophy type 1, 43.7% higher (p<0.0001) in obesity, and 11.9% higher (p=0.0029) in prediabetes. However, Clec5a expression is lower (P=0.0021) in Duchenne muscular dystrophy and Obesity-type 2 diabetes mellitus (p<0.0001) (**Figure 5F**).

The pancreas is a common site of disease in the digestive system, and several common afflictions include cystic fibrosis, pancreatic cancer, and pancreatitis^43^. **Figure 5G** shows that expression of Clec5a is lower in pancreas adenocarcinoma (PAAD), pancreatic cancer, pancreatic carcinoma (p<0.0001), and there is no difference in the pancreas in different types of diabetes mellitus (**Figure 5G**).

**Figure 6:**
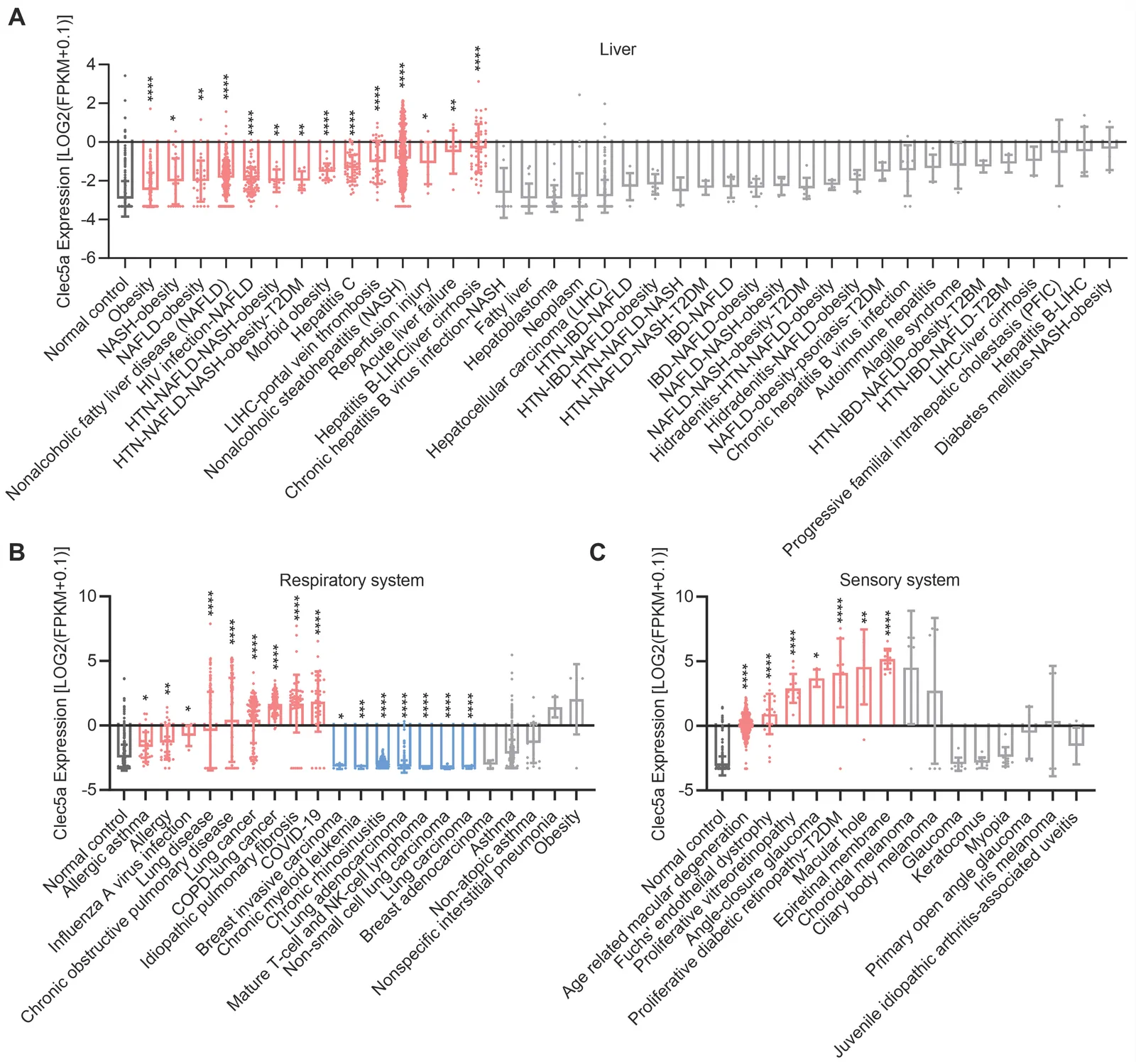
** Clec5a expression in human diseases**, including (**A**) liver, (**B**) respiratory system, (**C**) sensory system. Original data of RNA-seq or scRNA-seq for Clec5a expression, quantified by Log2 (FPKM + 0.1), were downloaded from QIAGEN OmicSoft Land Explorer. The data were analyzed with GraphPad Prism 10.1.2 and shown as the mean ± SD. Dunnett's one-way ANOVA was used for multiple comparisons between disease types and normal control. P < 0.05 was considered statistically significant.

Liver diseases involve metabolic, inflammatory, infectious, and neoplastic conditions. We found that Clec5a is extensively upregulated in a variety of liver diseases such as acute liver failure, chronic hepatitis B virus infection, hepatitis C, obesity, nonalcoholic fatty liver disease (NAFLD), nonalcoholic steatohepatitis (NASH), NASH-obesity, reperfusion injury (**Figure 6A**). Respiratory diseases affect airways and lung parenchyma. We found that Clec5a is significantly upregulated in allergies, COPD, COPD-lung cancer, lung cancer, lung disease, idiopathic pulmonary fibrosis, COVID-19. In contrast, Clec5a expression was 27.5% lower (P<0.0001) in lung adenocarcinoma (**Figure 6B**). The sensory system is part of the nervous system that uses specialized receptors to detect external or internal stimuli and convert them into suitable physiological responses. The RNA-seq data showed that, compared with normal control, Clec5a is extensively upregulated in a variety of sensory system diseases such as age-related macular degeneration, epiretinal membrane, Fuchs' endothelial dystrophy, and macular hole (**Figure 6C**).

**Figure 7:**
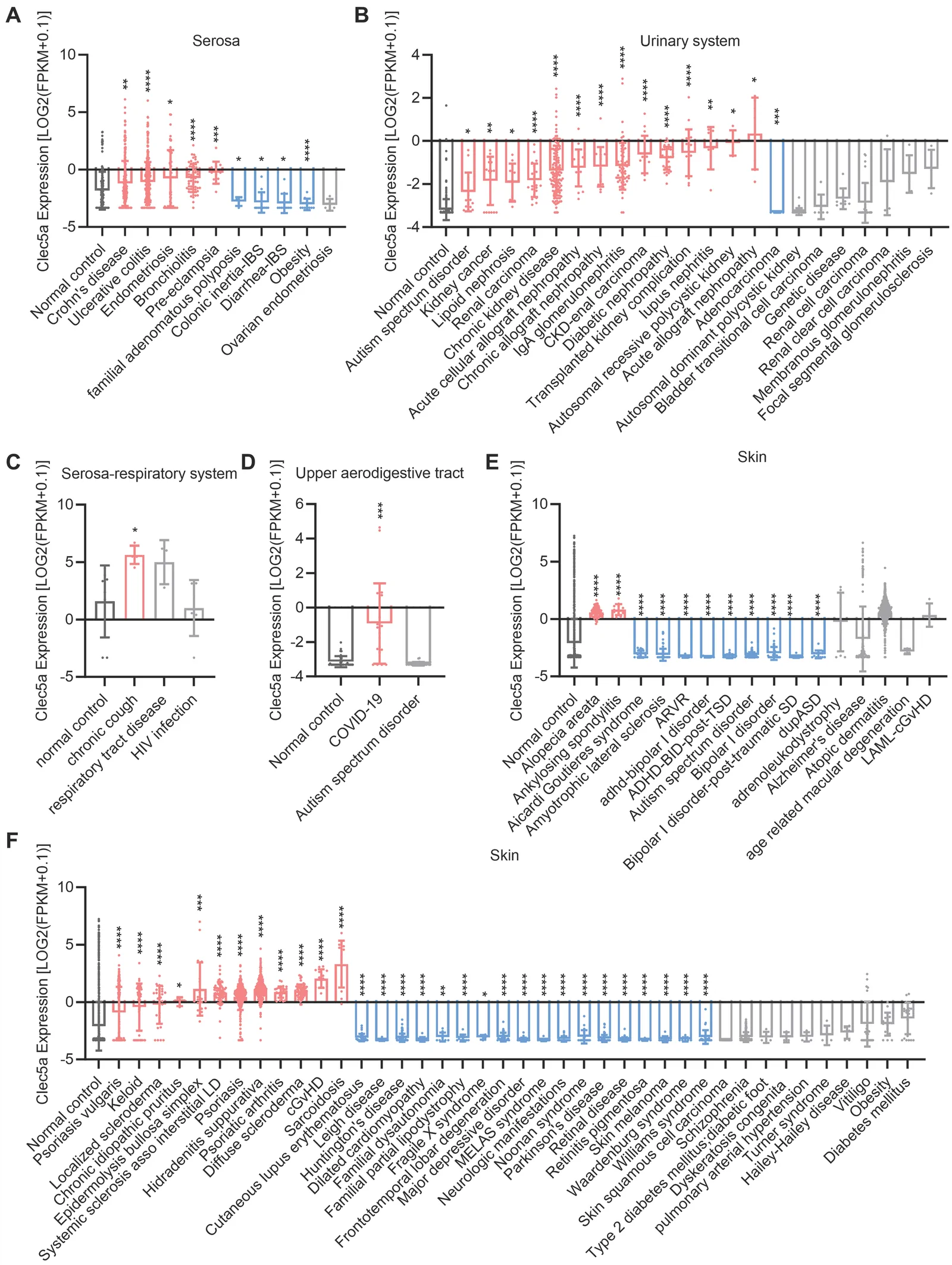
** Clec5a expression in human diseases** specifically in (**A**) serosa, (**B**) urinary system, (**C**) serosa-respiratory system, (**D**) upper aerodigestive tract, and (**E-F**) skin. Original data of RNA-seq or scRNA-seq for Clec5a expression, quantified by Log2 (FPKM + 0.1), were downloaded from QIAGEN OmicSoft Land Explorer. The data were analyzed with GraphPad Prism 10.1.2 and shown as the mean ± SD. Dunnett's one-way ANOVA was used for multiple comparisons between disease types and normal control. P < 0.05 was considered statistically significant.

Serosa consists of an epithelial layer and a connective tissue layer and can facilitate lubrication between organs by secreting serous fluid. Our RNA-seq big data analytics showed that, compared with normal control, Clec5a expression is significantly higher in many serosa disorders including bronchiolitis, Crohn's disease, endometriosis, ulcerative colitis, pre-eclampsia (**Figure 7A**). Common disorders of the urinary system include nephrosis (a noninflammatory kidney condition), nephrolithiasis (kidney stone formation), urethritis (inflammation of the urethra), and symptoms such as nocturia and enuresis^44^. We found that Clec5a is highly expressed in many urinary system-related diseases such as autism spectrum disorder, kidney cancer, lupus nephritis, renal carcinoma, chronic kidney disease, and lipoid nephrosis (**Figure 7B**). Serosa is also a key factor of respiratory system. Interestingly, our data showed that Clec5a is highly activated in chronic cough (**Figure 7C**). Similarly, Clec5a expression is 70.6% higher (P<0.001) compared to normal control in COVID-19 related upper aerodigestive tract diseases (**Figure 7D**).

As the largest organ, the skin exhibits diverse disease patterns. We analyzed Clec5a expression in skin and found that Clec5a is extensively upregulated in a variety of skin diseases such as alopecia areata, ankylosing spondylitis, hidradenitis suppurativa, psoriasis, psoriatic arthritis, sarcoidosis, and diffuse scleroderma. Our data showed that Clec5a expression is downregulated in amyotrophic lateral sclerosis, autism spectrum disorder, Huntington's disease, Parkinson's disease, retinal disease (**Figure 7E-F**).

### Sex differences of Clec5a expression in humans

**Figure 8:**
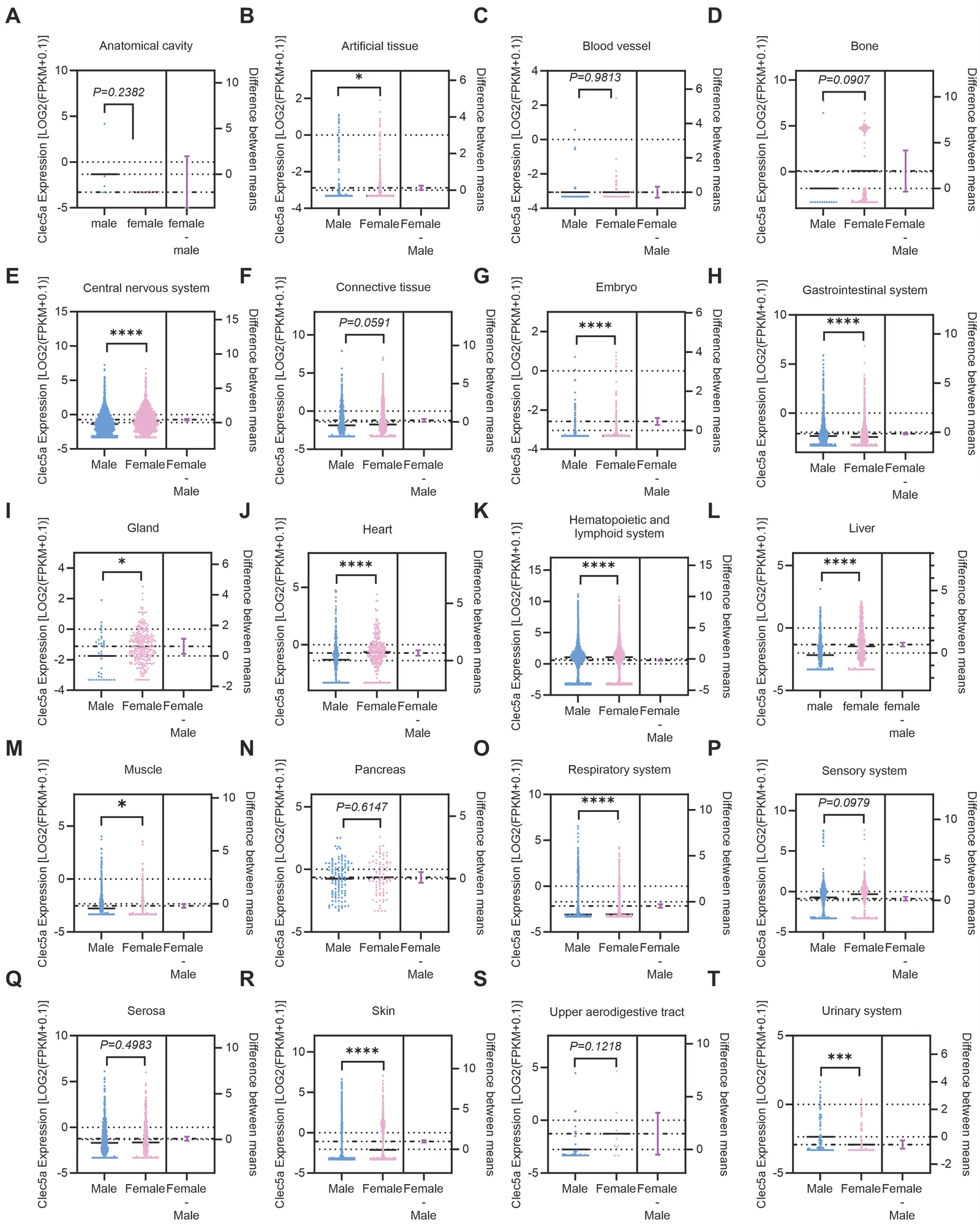
** Clec5a expression in human tissues from male and female**, including (**A**) anatomical cavity, (**B**) artificial tissue, (**C**) blood vessel, (**D**) bone, (**E**) central nervous system, (**F**) connective tissue, (**G**) embryo, (**H**) gastrointestinal system, (**I**) gland, (**J**) heart, (**K**) hematopoietic and lymphoid system, (**L**) liver, (**M**) muscle, (**N**) pancreas, (**O**) respiratory system, (**P**) sensory system, (**Q**) serosa, (**R**) skin, (**S**) upper aerodigestive tract, and (**T**) urinary system. Original data of RNA-seq or scRNA-seq for Clec5a expression, quantified by Log2 (FPKM + 0.1), were downloaded from QIAGEN OmicSoft Land Explorer. The data were analyzed with GraphPad Prism 10.1.2 and shown as the mean ± SD. Differences between male and female groups were analyzed using unpaired Student's t-tests. P < 0.05 was considered statistically significant.

In the reproductive system, we have observed an interesting finding for a significant difference in Clec5a expression between males and females (**Figure 3I**). We thereby hypothesized that Clec5a expression in other tissues may differ significantly between male and female. To test this hypothesis, we performed the fourth big data analytics based on RNA-seq database from Qiagen IPA. Our data showed that there is no difference between male and female for Clec5a expression in tissues of anatomical cavity (**Figure 8A**), blood vessel (**Figure 8C**), bone (**Figure 8D**), connective tissues (**Figure 8F**), pancreas (**Figure 8N**), sensory system (**Figure 8P**), serosa (**Figure 8Q**), and upper aerodigestive tract (**Figure 8S**). However, Clec5a expression is 7.4% higher (P<0.0001) in males than females in gastrointestinal system (**Figure 8H**), 34.8% higher (P<0.0001) in hematopoietic and lymphoid tissues (**Figure 8K**), 7.7% higher (P=0.0255) in muscle (**Figure 8M**), 21.5% higher (P<0.0001) in respiratory system (**Figure 8O**), 19.6% higher (P<0.001) in urinary system (**Figure 8T**). Meanwhile, we found that Clec5a expression is 4.4% lower (P=0.0282) in male than female in artificial tissues (**Figure 8B**), 41.7% lower (P<0.0001) in CNS (**Figure 8E**), 17.9% lower (P<0.0001) in embryo (**Figure 8G**), 55.5% lower (P=0.0152) in gland (**Figure 8I**), 92.5% lower (P<0.0001) in heart (**Figure 8J**), 51.2% lower (P<0.0001) in liver (**Figure 8L**), 90.9% lower (P<0.0001) in skin (**Figure 8R**). These findings suggest that the characteristics of Clec5a expression with sex differences need to be considered for therapeutic strategies to treat Clec5a-mediated human diseases.

**Figure 9:**
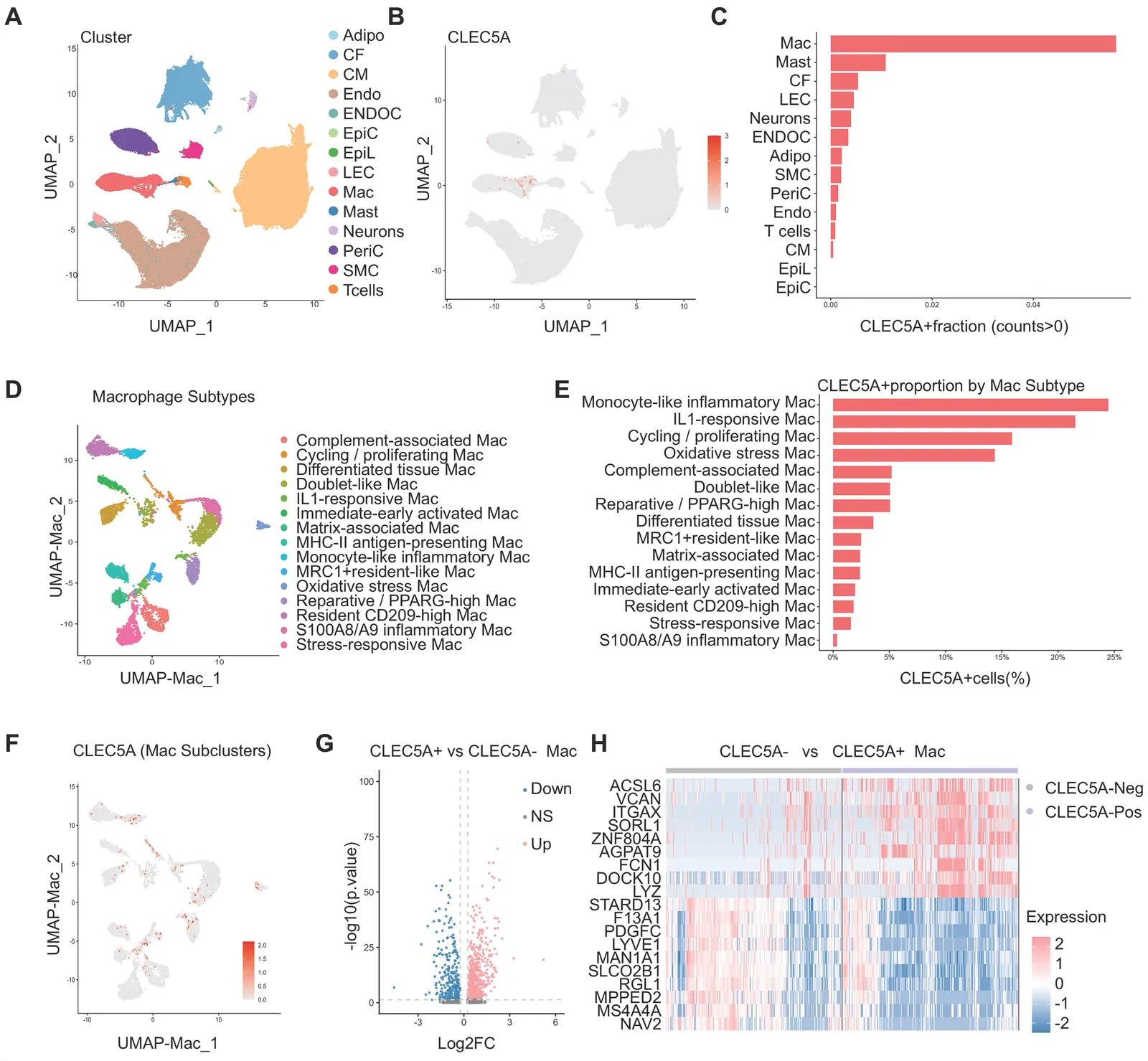
** SnRNA-seq for Clec5a dynamics in human tissues,** including (**A**) UMAP colored by annotated major cell types, (**B**) CLEC5A feature plot, (**C**) proportion of CLEC5A-positive cells across major cardiac cell types, (**D**) UMAP visualization of macrophage subclusters identified by reclustering of the myeloid compartment, (**E**) CLEC5A feature plot (macrophage subclusters), (**F**) proportion of CLEC5A-positive cells across macrophage subtypes, (**G**) CLEC5A-positive vs CLEC5A-negative macrophage DEG, and (**H**) heatmap. Abbreviations: Adipo: adipocytes; CF: cardiac fibroblasts; CM: cardiomyocytes; Endo: endothelial cells; ENDOC: endocardial cells; EpiC: epicardial cells; EpiL: epicardial-like cells; LEC: lymphatic endothelial cells; Mac: macrophages; Mast: mast cells; PeriC: pericytes; SMC: smooth muscle cells; UMAP: Uniform Manifold Approximation and Projection; DEG, differentially expressed genes; Pos: positive; Neg: negative; NS: Not significant; Log2FC: Log2 fold change.

**Sing-cell analysis for functions and mechanisms of CLEC5A-positive macrophages in congenital heart disease.** One of our interesting findings is that, among all the immune cells, CLEC5A has the highest expression in macrophages (**Figure 2A**). Focusing on CLEC5A expression in macrophages, we further analyzed a shared snRNA-seq data in the heart^21^, the first organ to develop and its defection leads to congenital heart disease^45^. In this snRNA-seq data, we for the first time separated CLEC5A-positive macrophages in 157,273 nuclei from control hearts and hearts from patients with different congenital heart diseases, including hypoplastic left heart syndrome, tetralogy of Fallot, cyanotic congenital heart disease lesions, dilated cardiomyopathies, and hypertrophic cardiomyopathies. To define the underlying cellular context and mechanisms of CLEC5A in congenital heart disease, we first summarized the unsupervised clustering of all heart cells. As shown in **Figure 9A**, we identified 14 major cardiac cell populations with unsupervised clustering, including cardiomyocytes, cardiac fibroblasts, endothelial cells, endocardial cells, epicardial cells, epicardial-like cells, lymphatic endothelial cells, smooth muscle cells, pericytes, adipocytes, neurons, macrophages, mast cells, and T lymphocytes. Interestingly, feature plot visualization demonstrated that CLEC5A expression is highly restricted to immune cell compartments, with predominant enrichment in macrophages (**Figure 9B**). Quantitative analysis confirmed that macrophages exhibited the highest fraction of CLEC5A-positive cells among all major cell types, whereas other populations showed minimal expression of CLEC5A (**Figure 9C**). To characterize CLEC5A expression within the immune cell compartments, macrophages were subclustered into transcriptionally distinct subtypes. Our data showed that CLEC5A expression is not only uniformly distributed across macrophage populations but also is preferentially enriched in specific inflammatory and stress-associated macrophage subtypes, including monocyte-like inflammatory macrophages and IL1-responsive macrophages (**Figure 9D-F**). These findings indicate that CLEC5A represents a novel marker for a defined subset of macrophages rather than representing a general macrophage marker.

Next, we compared transcriptional profiles between CLEC5A-positive and CLEC5A-negative macrophages to define functional programs associated with CLEC5A expression. Differential expression analysis identified many genes significantly changed in CLEC5A-positive macrophages (**Figure 9G-H**). Notably, CLEC5A-positive macrophages showed higher expression of inflammatory and myeloid-associated genes, including ACSL6, VCAN, FCN1, DOCK10, and LYZ, whereas CLEC5A-negative macrophages were characterized by relatively higher expression of genes associated with tissue-resident and vascular-related macrophage features, such as LYVE1, PDGFC, MS4A4A, and STARD13.

**Figure 10:**
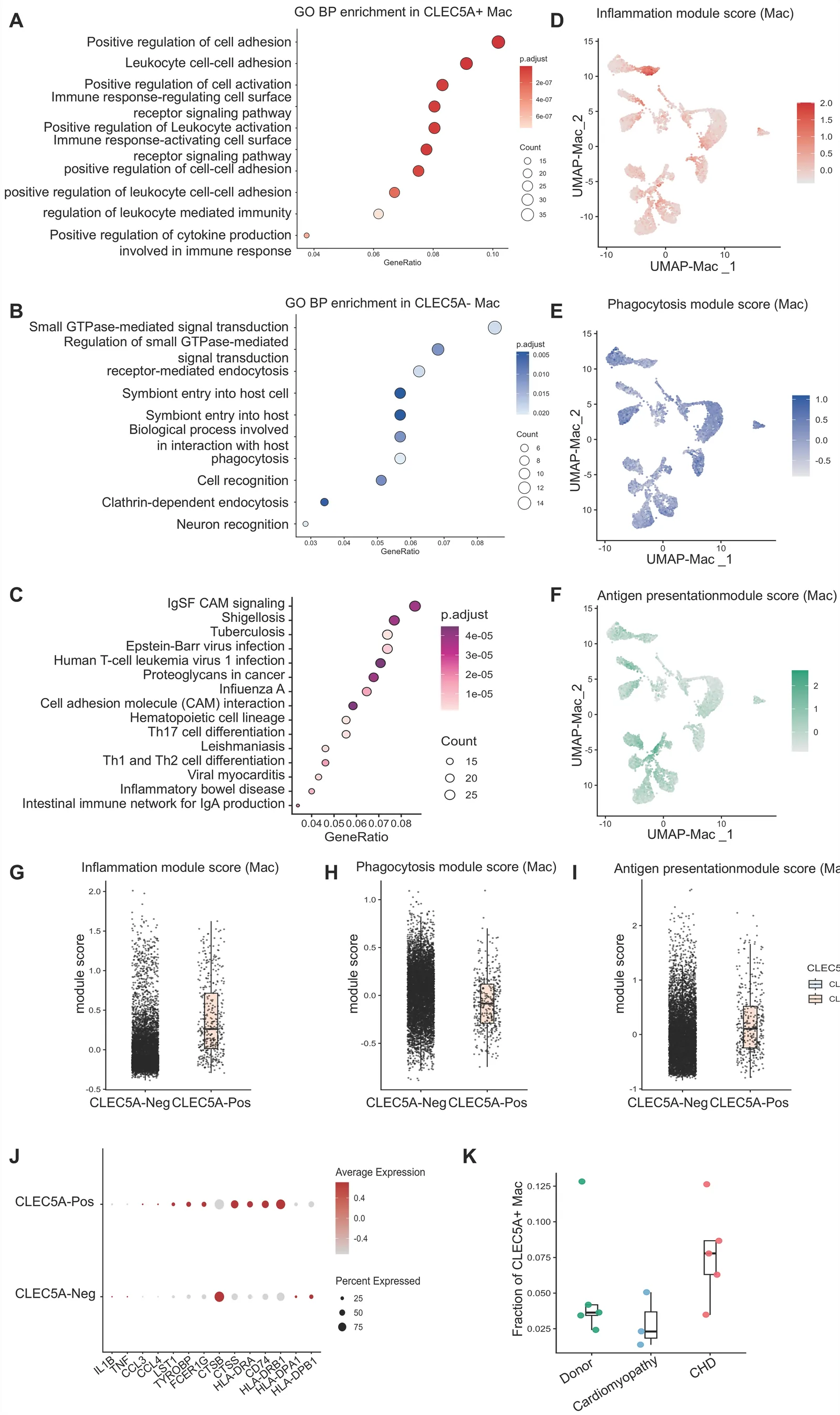
** Functional of CLEC5A-positive macrophages in human congenital heart disease,** including (**A**) GO BP enrichment analysis of genes upregulated in CLEC5A-positive macrophages, (**B**) GO BP enrichment analysis of genes enriched in CLEC5A-negative macrophages, (**C**) KEGG pathway enrichment analysis of genes upregulated in CLEC5A-positive macrophages, (**D-F**) UMAP visualization of module scores for inflammation, phagocytosis, and antigen presentation in macrophages, demonstrating spatial enrichment of these functional signatures in CLEC5A-positive macrophages, (**G-I**) Box plots comparing inflammation, phagocytosis, and antigen presentation module scores between CLEC5A-negative and CLEC5A-positive macrophages, (**J**) Dot plot showing expression of representative inflammatory and antigen presentation marker genes in CLEC5A-negative and CLEC5A-positive macrophages, (**K**) Fraction of CLEC5A-positive macrophages across different disease conditions, including donor controls, cardiomyopathy, and CHD. Abbreviations: GO BP: Gene Ontology Biological Process; KEGG: Kyoto Encyclopedia of Genes and Genomes; UMAP: Uniform Manifold Approximation and Projection; Mac: macrophages; Pos: positive; Neg: negative; CHD: congenital heart disease.

To further characterize transcriptional programs associated with CLEC5A expression in macrophages, we performed functional enrichment analyses. Gene Ontology Biological Process (GO BP)^46^ analysis revealed that genes upregulated in CLEC5A-positive macrophages were significantly enriched in immune activation-related processes, including leukocyte activation, cytokine production, cell-cell adhesion, and innate immune signaling (**Figure 10A**). In contrast, genes enriched in CLEC5A-negative macrophages were primarily associated with small GTPase mediated signal transduction, endocytic processes, and cell recognition pathways, reflecting distinct functional states of macrophages lacking CLEC5A expression (**Figure 10B**).Consistent with the GO BP results observed in CLEC5A-positive macrophages, KEGG pathway analysis^47^ further demonstrated an enrichment of immune- and inflammation-related pathways, including cell adhesion molecule (CAM) signaling, Th1 and Th2 cell differentiation, and infection-associated pathways (**Figure 10C**). Consistent with these results, module score analyses demonstrated that CLEC5A-positive macrophages exhibited significantly higher inflammation, phagocytosis, and antigen presentation scores compared with CLEC5A-negative macrophages (**Figure 10D-I**). These functional signatures spatially overlapped with CLEC5A expression on UMAP projections and were quantitatively elevated in CLEC5A-positive macrophages, indicating that CLEC5A expression reflects a stable inflammatory activation state. Expression of canonical inflammatory and antigen presentation markers was coordinately increased in CLEC5A-positive macrophages (**Figure 10J**), further supporting the concept that CLEC5A represents a transcriptionally and functionally distinct macrophage state. Importantly, the proportion of CLEC5A-positive macrophages varied across donors and disease conditions but remained consistently associated with inflammatory macrophage phenotypes (**Figure 10K**).

Together, this snRNA-seq analysis highlighted the importance of CLEC5A in the identification of macrophages subtypes characterized by enhanced inflammatory signaling, phagocytic capacity, and antigen presentation activity. These findings provide deep cellular and functional viewpoints for CLEC5A in special human disorders of congenital heart disease based on bulk transcriptomic observations, establishing CLEC5A as a marker of pathogenic macrophage activation rather than a broadly expressed gene in immune cells.

**Figure 11:**
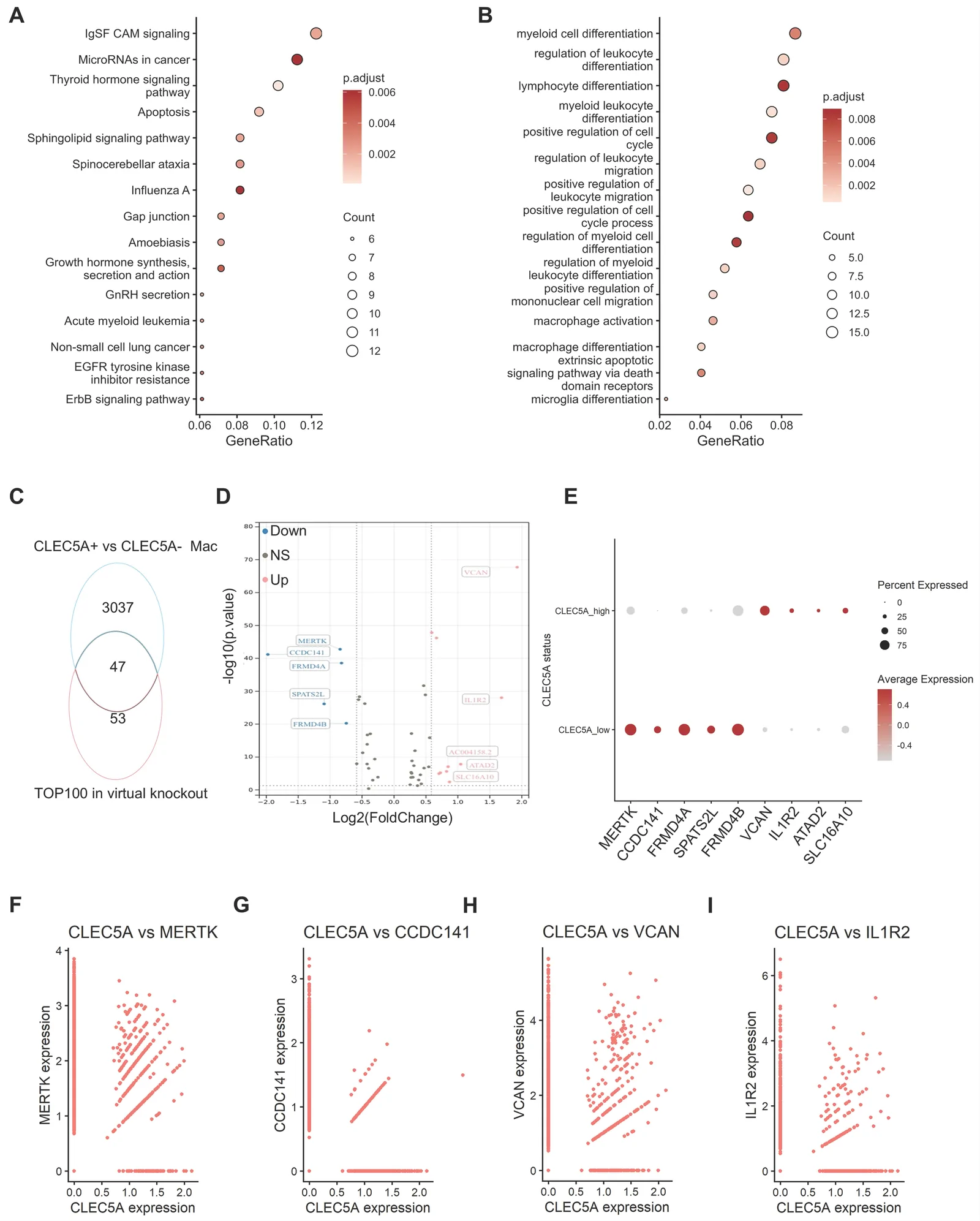
***In silico* virtual CLEC5A knockout in human macrophages,** including (**A**) KEGG pathway enrichment analysis of genes affected by *in silico* virtual knockout of CLEC5A in macrophages, (**B**) GO BP enrichment analysis of genes affected by virtual CLEC5A knockout, (**C**) Venn diagram showing the overlap between differentially expressed genes (DEGs) identified in CLEC5A-positive versus CLEC5A-negative macrophages and the top genes perturbed by virtual CLEC5A knockout, (**D**) volcano plot illustrating transcriptional changes following virtual CLEC5A knockout, (**E**) dot plot showing expression patterns of representative overlapping genes between CLEC5A-positive macrophage DEGs and virtual knockout-perturbed genes across CLEC5A-high and CLEC5A-low macrophages, (**F-I**) correlation analyses between CLEC5A expression and selected downstream genes, including MERTK, CCDC141, VCAN and IL1R2. Abbreviations: KEGG: Kyoto Encyclopedia of Genes and Genomes; GO BP: Gene Ontology Biological Process; DEG: differentially expressed gene; Mac: macrophages; Pos: positive; Neg: negative.

***In silico* virtual knockout analyses to validate the role of CLEC5A in macrophage activation.** *In silico* virtual knockout analysis is a novel approach using computational models that are often based on single-cell RNA sequencing data to simulate gene knockout to predict its function and biological effects^22^. Therefore, we performed *in silico* virtual CLEC5A knockout analyses to simulate the transcriptional consequences of CLEC5A knockout and evaluate the potential functions of CLEC5A in macrophage activation. Genes exhibiting the most significant predicted expression changes following virtual CLEC5A knockout were subjected to pathway enrichment analyses. KEGG pathway represents the knowledge of molecular interaction, reaction and relation networks. Our KEGG pathway analysis revealed that CLEC5A virtual knockout-mediated genes were significantly enriched in immune- and inflammation-related signaling pathways, including cell adhesion molecule signaling, apoptosis, sphingolipid signaling, and infection-associated pathways (**Figure 11A**). These results suggest that CLEC5A virtual knockout selectively impacts signaling programs involved in immune cell communication, inflammatory responses, and host defense. Consistent with these findings, GOBP enrichment analysis demonstrated strong overrepresentation of pathways related to myeloid cell differentiation, regulation of leukocyte differentiation, macrophage activation, and mononuclear cell migration among genes affected by CLEC5A virtual knockout (**Figure 11B**). These enriched biological processes closely mirror the transcriptional programs identified in CLEC5A-positive macrophages in the single-cell analyses, indicating a coherent functional link between CLEC5A expression and macrophage inflammatory states.

To directly link the virtual knockout predictions with the CLEC5A-positive macrophages, we intersected the top 100 genes showing the largest predicted expression changes, following CLEC5A knockout with genes differentially expressed between CLEC5A-positive and CLEC5A-negative macrophages. This analysis identified a shared set of 47 genes (**Figure 11C**), the majority of which were upregulated in CLEC5A-positive macrophages. Volcano plot highlighted representative overlapping genes with the strongest predicted downregulation or upregulation upon CLEC5A knockout (**Figure 11D**). Notably, several downregulated genes (e.g., MERTK, CCDC141, FRMD4A, SPATS2L, and FRMD4B) are associated with macrophage function and cellular regulation^48^,^49^, whereas selected upregulated genes such as VCAN and IL1R2 may reflect compensatory or secondary transcriptional responses to CLEC5A virtual knockout. Expression patterns of these representative genes further demonstrated clear stratification between CLEC5A-positive and CLEC5A-negative macrophages, as visualized by dot plot analysis (**Figure 11E**). In addition, correlation analyses confirmed the associations between CLEC5A expression and selected downstream genes at the single-cell level (e.g, MERTK, CCDC141, VCAN and IL1R2, **Figure 11F-I**), supporting coordinated regulation within CLEC5A-associated transcriptional programs.

Together, our virtual gene knockout analytics supports a model by which CLEC5A contributes to the maintenance of inflammatory macrophages and differentiation-associated macrophage transcriptional programs. Basic experiments validation may be required to establish the causality of CLEC5A in macrophage function, however, the concordance between virtual knockout predictions and single-cell expression features support that CLEC5A occupies a crucial functional role in immune regulatory networks in macrophages.

## Discussion

CLEC5A is a crucial innate immune receptor on myeloid cells (e.g., macrophages, monocytes, and neutrophils) that detects pathogens and subsequently triggers inflammation and immune responses^50^, playing important roles in a variety of human diseases including cardiovascular diseases, cancer and brain disorders. Combined with state-of-the-art techniques such as RNA-seq big data analytics, proteomics big data analytics, snRNA-seq, and *in silico* virtual knockout analyses, we for the first time provided a systematic analysis of Clec5a functions in a wide range of human cells, tissues and diseases^22^,^23^,^27^. Our big data analytics indicates that Clec5a is strongly enriched in myeloid lineage compartments and has a context dependent dysregulation across inflammatory, cardiovascular, metabolic, hepatic, renal, neurologic and cutaneous disorders. We also identified developmental and sex related differences for Clec5a biology, suggesting that life stage and biological sex have important impact of Clec5a functions on innate immune responses in humans.

At the cellular level, Clec5a is mainly expressed in macrophages, monocytes, dendritic cells and neutrophil progenitors, while most mesenchymal stromal cells, epithelial cells and neural cells show minimal transcript levels. This pattern is consistent with previous study that defined CLEC5A as a myeloid DAP12 associating lectin, which amplifies Syk dependent signaling and promotes production of proinflammatory cytokines in macrophages and neutrophils during infection and sterile injury^3^. The enrichment of Clec5a in innate immune cells, and its low expression in structural and parenchymal cells, shows the concept that CLEC5A functions primarily as an amplifier of myeloid driven inflammation rather than a broad sensor in all tissue compartments^51^,^52^. Here, we use the term of CLEC5A-associated hyperinflammation to describe a dysregulated myeloid-centered inflammatory state marked by excessive cytokine and chemokine production, inflammasome activation, neutrophil extracellular trap formation, vascular leakage, and tissue injury^15^. Importantly, evidence from viral infection, autoimmune inflammation, chronic pulmonary disease, and cardiovascular injury indicates that CLEC5A may contribute to multi-organ immunopathology with the heart representing as a clinically relevant organ. Our tissue data analyses further extended these cellular insights and showed that Clec5a transcripts are detectable in many adult organs but generally remain at a low level, with notable exceptions in plaque and serosa related tissues. These findings align with reports that CLEC5A is highly expressed in lesional macrophages in humans and is induced by proatherogenic stimuli such as oxidized LDL and hypoxia. In vascular and serosal beds that are constantly exposed to mechanical and inflammatory stress, elevated Clec5a expression may promote local production of cytokines and chemokines, enhance leukocyte recruitment, and thereby contribute to plaque progression, vascular inflammation and serosal injury^20^. At the same time, the low expression observed in many other tissues suggests that CLEC5A can be rapidly mobilized when myeloid cells infiltrate or become activated in these sites.

Clec5a dysregulation in tissue specific diseases shows several recurring patterns. First, Clec5a tends to be increased in conditions of chronic inflammation or barrier disruption, including inflammatory bowel disease, chronic liver disease, chronic kidney disease, chronic obstructive pulmonary disease, asthma, autoimmune skin diseases and several neuroinflammatory disorders. In these diseases, CLEC5A may act as an innate immune checkpoint that amplifies danger signals and sustains myeloid activation, like what has been described in viral infections and arthritis^8^. Conversely, Clec5a expression is reduced in selected malignant or degenerative conditions such as pancreatic cancers in our dataset. Whether this decrease of CLEC5A reflects immune escape, loss of proinflammatory myeloid populations, or tissue remodeling remains unclear and will require functional validation. Together, these findings suggest that Clec5a activation is highly associated with inflammatory and fibrotic pathology, whereas Clec5a deficiency may accompany neoplastic or degenerative states.

Our big data analytics also highlighted the importance of Clec5a in cardiovascular and cardio-metabolic diseases. Clec5a expression is elevated in atherosclerotic plaque and multiple cardiovascular tissue specific disorders, and basic studies have shown that CLEC5A promotes macrophage survival, NLRP3 inflammasome activation and pyroptosis in post of myocardial infarction^36^. On the one hand, our findings support a novel concept in which CLEC5A sustains inflammatory macrophage phenotypes in the heart and vasculature, thereby contributing to impaired inflammation resolution and adverse cardiac remodeling. One the other hand, due to CLEC5A is also required for host defense against viral infection^10^, complete CLEC5A blockage may increase the risk of infection related disorders. Future studies need to define the quantity and timing of CLEC5A blockade that can maximally attenuate chronic vascular inflammation while preserving adequate antimicrobial immunity.

Developmental comparisons between embryonic and adult tissues show that Clec5a expression is generally lower in embryonic blood vessels, connective tissue, central nervous system and respiratory system than in their adult counterparts, whereas hematopoietic and lymphoid tissues demonstrate similar levels. These data suggest that Clec5a driven inflammatory amplification is relatively muted in fetal structural tissues, which may protect developing organs from excessive immune response-mediated tissue damage. During the process of organ development, hematopoietic tissues may be already adapted to Clec5a dependent immune responses. Our analysis also identified tissue-specific sex differences in CLEC5A expression. In tissues including the central nervous system, gland, heart, liver, and skin, CLEC5A expression was higher in females than in males. In contrast, higher CLEC5A expression in males was observed in the gastrointestinal system, hematopoietic and lymphoid system, muscle, respiratory system, and urinary system. These findings suggest that sex-dependent CLEC5A regulation is not restricted to a single organ but may represent a broader tissue specific feature of innate immune regulation. Sex hormones may be one potential mechanism underlying these differences^53^. Estrogen and androgen signaling can differentially regulate macrophage polarization, cytokine production, inflammasome activation, and tissue immune homeostasis in a context-dependent manner^54^. Thus, estrogen-related immune modulation may contribute to the enrichment or activation of CLEC5A-positive myeloid cells in selected female tissues^55^, whereas androgen-related or tissue-specific inflammatory programs may contribute to higher CLEC5A expression in selected male tissues. However, our current analysis is based on transcriptomic datasets, future studies using sex-balanced cohorts or male/female animal models are recommendated to determine whether CLEC5A directly contributes to sex-dependent inflammatory responses in cardiovascular and other inflammatory diseases.

Our snRNA-seq analysis further demonstrated that CLEC5A is a novel marker for proinflammatory macrophages with specialized functional characteristics^21^. CLEC5A-positive macrophages were found to be enriched for genes and pathways related to cytokine production, phagocytosis, and antigen presentation, indicating that CLEC5A expression delineates a highly activated inflammatory macrophage state. This finding aligns with CLEC5A's known role as an amplifier of innate immune responses and provides cellular-level context by aiming the myeloid population it influences. Moreover, the virtual CLEC5A knockout analysis supports a causal network for CLEC5A in sustaining inflammatory programs in macrophages^22^. The virtual gene deletion predicted a variety of down-regulators for immune and inflammatory signaling pathways. Notably, many of the genes identified as perturbed by CLEC5A knockout overlapped with those upregulated in CLEC5A-positive macrophages, emphasizing a coherent functional link between CLEC5A and macrophage activation. Together, the evidence of single-cell evidence and network-based predictions highlights CLEC5A as a novel node for regulatory circuits that drive inflammation in macrophages, reinforcing its immune related functional role in human diseases.

The present study also highlights the value of big data analytics in humans as a bridge between clinical observation and experimental validation. Unlike hypothesis-driven studies focused on a single disease model, integrative human datasets allow systematic evaluation of gene expression across multiple organs, disease states, biological sex, developmental stages, and cell types. This strategy can identify clinically relevant disease contexts, define target cell populations, and guide prioritization of therapeutic indications before entering animal experiments. For preclinical drug design, such information is valuable for selecting the most appropriate disease models, predicting potential tissue-specific effects, and identifying patient populations that may benefit from CLEC5A-targeted interventions^56^. For mechanistic studies, human data can generate focused hypotheses that are subsequently tested in animals using conditional gene deletion, receptor blockade, or pathway inhibition^57^. Our findings provide a strong rationale to use macrophage-specific CLEC5A gene knockout/overexpression in models of inflammation-associated diseases.

From a translational perspective, our big data analytics provides the first evidence that CLEC5A can be considered as a novel biomarker and therapeutic target in inflammation related human diseases, including cardiovascular diseases, cancer and brain disorders. Clec5a level measurement in tissues or circulating cell populations, such as monocytes or lesional macrophages in blood, may help identify excessive innate immune activation in patients who may benefit from CLEC5A blockade treatment. However, we acknowledge that Clec5a level quantification in tissues may not be stable and require alternative measurement strategies. Current therapeutic strategies targeting CLEC5A-associated hyperinflammation remain largely to be identified and validated. The most direct approach is receptor blockade using antagonistic anti-CLEC5A monoclonal antibodies, which have been reported in experimental infection and inflammatory disease models to reduce cytokine production, inflammasome activation, vascular leakage, and tissue injury^5^,^6^,^7^. Additional strategies include receptor-decoy approaches, inhibition of the CLEC5A-DAP12-Syk signaling axis, and combined blockade of CLEC5A with other cooperating innate immune receptors, such as TLR2^58^. Because CLEC5A functions upstream of several inflammatory pathways, downstream anti-inflammatory strategies targeting IL-1β, IL-6, JAK/STAT, or NLRP3 inflammasome signaling may also be conceptually relevant, although these approaches are not CLEC5A-specific. Importantly, clinically established CLEC5A-blocking therapies are not yet available, and future studies are needed to determine whether selective CLEC5A inhibition can suppress pathological inflammation while preserving host defense.

Several limitations of our study are summarized as follows. First, our big data analytics are mainly based on bulk RNA-seq, snRNA-seq, and proteomics in human tissues. The big data analytics well capture both transcript abundance and protein levels, but not for receptor activation state or downstream functional consequences. Future studies will use macrophage specific CLEC5A conditional knockout mice, pharmacological inhibition, and disease specific animal models to experimentally validate the causal role of CLEC5A in macrophage activation and tissue injury. Interested genes will be further investigated for mechanism validations with chemical inhibitors for gene inhibition or adeno-associated viruses for gene overexpression. Second, the underlying cohorts are heterogeneous with respect to age, comorbidities and treatment that may influence observed expression patterns of CLEC5A in cells or tissues. Third, independent experiment validation using patient samples also needs to be performed, particularly in cardiovascular diseases models that have been studying in our groups, including atherosclerosis, myocardial infarction and vascular aging. The future work will integrate our findings with proteomics, spatial transcriptomics and functional assays in relevant models of cardiovascular diseases, chronic inflammation diseases (e.g. chronic liver disease, chronic kidney disease) and neuroinflammation. Deep analyses of Clec5a signaling networks in human myeloid subsets, including interactions with DAP12, Syk and inflammasome components, may identify key interact signaling that can be targeted more safely than Clec5a itself for clinical application. Together, our big data analytics firstly provide a framework for understanding how Clec5a level is distributed across human tissues and diseases. Our comprehensive genomic and proteomic analytics as well as specific snRNA-seq and *in silico* virtual knockout analytics also indicate a promising translational direction aiming at CLEC5A modulation in human diseases.

In summary, our comprehensive big data analysis compellingly establishes CLEC5A as a myeloid specific receptor broadly implicated in human diseases. CLEC5A is highly enriched in innate immune cells and shows dynamic regulation across numerous tissues and disease states such as breast and connective tissue, atherosclerosis and heart failure, with notable developmental and sex-related differences. Our single-cell transcriptomic profiling clarified that CLEC5A is a novel marker for a distinct subset of proinflammatory macrophages, indicating CLEC5A as a promising biomarker and therapeutic target in hyperinflammatory conditions.

## Methods

**The Human Protein Atlas.** To characterize CLEC5A expression across human tissues and cell types, we utilized datasets from The Human Protein Atlas (HPA), a publicly available Swedish-based resource. HPA integrates multiple omics platforms, including antibody-based imaging, transcriptomics, and proteomics, to provide a genome-wide expression landscape. Specifically, we extract transcriptomic and proteomic data relevant to CLEC5A in diverse biological systems, including protein structure, protein expression, brain expression, and cancer-related expression data, were extracted from the corresponding HPA pages. These datasets facilitated cross-tissue comparisons and cell-type-specific resolution of CLEC5A distribution. All data used was retrieved from the open-access HPA portal (www.proteinatlas.org)^23^. Downloaded tables were manually curated to retain CLEC5A-specific entries and standardize group names for visualization. Processed data were imported into GraphPad Prism 10.1.2 for statistical analysis and figure generation.

**QIAGEN Ingenuity Pathway Analysis (IPA).** CLEC5A was searched using the official gene symbol, and expression datasets were filtered to include human samples with available metadata for cell type, tissue source, disease state, developmental stage, and sex. For cell-type analyses, CLEC5A expression values were extracted from available RNA-seq or single-cell RNA-seq summary datasets and grouped by annotated cell identity. For tissue, disease, developmental, and sex stratified analyses, CLEC5A expression values were extracted from human RNA-seq datasets and grouped according to the corresponding OmicSoft metadata fields, including Sample Source, Tissue, Disease State, Development Stage, and Sex. Expression levels were exported from OmicSoft Land Explorer as normalized values, including nCPM for cell-type summaries and log2(FPKM + 0.1) for tissue and disease comparisons, as indicated in the corresponding figure legends. Exported tables were manually reviewed to ensure that group labels matched the categories shown in each figure. Samples lacking the required metadata for a specific comparison were excluded from that specific analysis. Data were then imported into GraphPad Prism 10.1.2 for statistical analysis and plotting. For two-group comparisons, unpaired Student's t-tests were used. For multiple disease groups compared with normal control, Dunnett's one-way ANOVA was used. Processed source data, group annotations, and values used for figure generation are provided in the Supplemental Data to improve transparency and reproducibility^26^.

**Single-nucleus RNA sequencing (snRNA-seq) in human congenital heart disease.** Single-nucleus RNA sequencing (snRNA-seq) data were obtained from a previously published study that performed integrated multi-omic profiling of pediatric congenital heart disease (CHD) hearts^21^. SnRNA-seq data was based on nine pediatric CHD heart samples and four donated control pediatric hearts (GSE203275), encompassing detailed profiles of 157,273 cardiac nuclei across 29,266 genes. The snRNA-seq data were processed using the Seurat pipeline (v4) for quality control, normalization, and unsupervised clustering. Data processing followed workflows consistent with the original study, with minor adaptations for downstream macrophage-focused analyses. Major cell types were annotated based on canonical markers, from which macrophages were identified as the primary myeloid population analyzed in this study. We reclustered macrophages to identify transcriptionally distinct macrophage subpopulations. Differential expression analysis (CLEC5A-positive vs CLEC5A-negative macrophages) was performed using Seurat's FindMarkers function (Wilcoxon rank-sum test), yielding a set of significantly up- and down-regulated genes associated with CLEC5A expression. These gene sets were subjected to Gene Ontology (GO) and Kyoto Encyclopedia of Genes and Genomes (KEGG) pathway enrichment analyses to characterize the biological processes and pathways overrepresented in CLEC5A-expressing macrophages.

***In silico* virtual gene knockout analyses**. For virtual functional analysis, we employed the scTenifoldKnk package to perform a virtual knockout of CLEC5A on the single cell data^22^. This machine-learning workflow constructs a single-cell gene regulatory network from the wild-type scRNA-seq expression matrix and then virtually removes the target gene by eliminating its network connections. The resulting “knockout” network is aligned and compared to the original network to identify differentially regulated genes (virtual knockout-perturbed genes) that represent the transcriptomic impact of CLEC5A loss. We applied scTenifoldKnk to the macrophage subset data with CLEC5A as the target and extracted the genes showing the most significant predicted changes upon CLEC5A removal. Pathway enrichment analysis (GO and KEGG) was then performed on these CLEC5A-perturbed genes to infer which cellular functions and signaling pathways would be most affected by the loss of CLEC5A. Finally, to integrate the predictions with our experimental observations, we compared the top CLEC5A knockout-perturbed genes to the CLEC5A-positive macrophage signature. The overlap between the virtual knockout targets and the CLEC5A^+^ vs CLEC5A^-^ macrophage differentially expressed genes (DEGs) was assessed, revealing a substantial intersection of genes and pathways. This comparative approach allowed us to validate the network model predictions against actual single-cell expression differences, strengthening the evidence for functional role of CLEC5A.

**Statistical analysis.** Differences between two groups were evaluated with unpaired Student's t-tests, and Dunnett's one-way ANOVA was applied for comparisons of multiple disease groups against normal control. Data were processed using GraphPad Prism 10.1.2 and are reported as mean ± SD. Statistical significance was defined as P < 0.05.
